# A Case of Transient Spontaneous Regression of Intrahepatic Cholangiocarcinoma Clinically Mimicking Inflammatory Pseudotumor: A Diagnostic Dilemma

**DOI:** 10.70352/scrj.cr.26-0248

**Published:** 2026-06-26

**Authors:** Shoya Yamaguchi, Tatsunori Miyata, Yoshiyuki Tagayasu, Shota Uemura, Yuta Shiraishi, Daisuke Ogawa, Shotaro Kinoshita, Takanobu Yamao, Yuki Kitano, Hirohisa Okabe, Keita Kai, Hiromitsu Hayashi, Masaaki Iwatsuki

**Affiliations:** 1 Department of Gastroenterological Surgery, Graduate School of Life Sciences, Kumamoto University, Kumamoto, Kumamoto, Japan; 2 Department of Diagnostic Pathology, Graduate School of Life Sciences, Kumamoto University, Kumamoto, Kumamoto, Japan

**Keywords:** intrahepatic cholangiocarcinoma, spontaneous regression, hepatic inflammatory pseudotumor

## Abstract

**INTRODUCTION:**

Spontaneous regression of intrahepatic cholangiocarcinoma (iCCA) is extremely rare and may create diagnostic confusion when mimicking benign inflammatory lesions. We herein report a case of iCCA that demonstrated transient spontaneous regression, which led to difficulty in differentiating it from hepatic inflammatory pseudotumor (HIPT).

**CASE PRESENTATION:**

A 70-year-old woman with alcoholic liver cirrhosis was found to have a 14-mm hepatic mass in segment 6 with peripheral ring-like enhancement on contrast-enhanced CT and high fluorodeoxyglucose (FDG) uptake (maximum standardized uptake value [SUVmax] 11.2). Carcinoembryonic antigen (CEA) and carbohydrate antigen 19-9 (CA19-9) were mildly elevated. iCCA, hepatocellular carcinoma, and combined hepatocellular–cholangiocarcinoma were considered. Two months later, the lesion regressed spontaneously to 5 mm without treatment, and CEA and CA19-9 decreased, suggesting a diagnosis of HIPT. However, the lesion gradually increased in size over the following year. CEA and CA19-9 subsequently increased markedly, and FDG uptake intensified (SUVmax 14.3). Given the strong suspicion of malignancy, laparoscopic partial hepatectomy was performed. Histopathological examination revealed an invasive iCCA with glandular formation, mucin production, necrosis with neutrophilic infiltration, and portal vein invasion.

**CONCLUSIONS:**

This case highlights that spontaneous tumor regression does not exclude malignancy. Careful long-term surveillance and timely surgical intervention are crucial when imaging findings and tumor marker trends raise suspicion of malignancy despite temporary regression.

## Abbreviations

CA19-9
: carbohydrate antigen 19-9
CEA
: carcinoembryonic antigen
cHCC-iCCA
: combined hepatocellular–cholangiocarcinoma
FDG-PET
: fluorodeoxyglucose PET
HCC
: hepatocellular carcinoma
HIPT
: hepatic inflammatory pseudotumor
iCCA
: intrahepatic cholangiocarcinoma
SUVmax
: maximum standardized uptake value

## INTRODUCTION

iCCA is the second most common primary liver malignancy and represents a highly aggressive tumor entity with increasing global incidence.^1)^ Despite advances in imaging modalities and molecular characterization, iCCA remains associated with a poor prognosis due to early vascular invasion and a desmoplastic tumor microenvironment that promotes immune evasion and tumor progression.^2,3)^ Mass-forming iCCA typically demonstrates progressive enlargement; therefore, spontaneous tumor regression is biologically unexpected.

Spontaneous regression of malignant tumors is an exceptionally rare phenomenon and is defined as partial or complete disappearance of a tumor without adequate treatment.^4)^ Although immune-mediated mechanisms and ischemic events have been implicated in spontaneous regression of several solid tumors, reports involving iCCA remain extremely limited.^4,5)^ When regression is observed, it is often transient and followed by tumor progression, suggesting incomplete tumor eradication rather than true biological remission.^5)^

A major diagnostic dilemma arises when iCCA exhibits atypical behavior mimicking benign inflammatory lesions. HIPT is a benign inflammatory mass characterized by fibroinflammatory proliferation and inflammatory cell infiltration.^6)^ HIPT frequently presents with imaging features overlapping those of iCCA, including peripheral ring-like enhancement on contrast-enhanced CT or MRI and increased uptake on FDG-PET.^6,7)^ Importantly, spontaneous regression is well documented in HIPT, and is often regarded as a characteristic clinical feature.^8)^ Consequently, regression of a hepatic mass may strongly favor an inflammatory diagnosis and lead to conservative management.

However, tumor shrinkage alone does not exclude malignancy. Here, we report a rare case of iCCA that demonstrated spontaneous regression without therapeutic intervention, closely mimicking HIPT, followed by tumor re-enlargement and pathological confirmation of invasive carcinoma with portal vein invasion. This case highlights the diagnostic pitfalls associated with transient tumor regression and underscores the importance of sustained vigilance when evaluating hepatic masses with fluctuating clinical behavior.

## CASE PRESENTATION

A 70-year-old woman with a history of alcoholic liver cirrhosis, esophageal varices, esophageal cancer, and cholelithiasis was regularly followed up after undergoing endoscopic submucosal dissection for esophageal cancer 5 years earlier at a previous hospital.

In November of year X–2, laboratory testing revealed elevated tumor markers (CEA 3.5 ng/mL and CA19-9 50.2 U/mL). CT demonstrated a 14-mm mass in segment 6 of the liver with peripheral ring-like enhancement (**Fig. 1A**–**1C**). Gadolinium ethoxybenzyl diethylenetriamine pentaacetic acid–enhanced MRI (EOB-MRI) showed similar peripheral enhancement, hypointensity in the hepatobiliary phase, and high signal intensity on diffusion-weighted imaging (**Fig. 1D**–**1F**). FDG-PET demonstrated abnormal uptake with an SUVmax of 11.2 (**Fig. 1G**). Based on these findings, HCC, iCCA, and cHCC-iCCA were considered in the differential diagnosis. The patient was therefore referred to our hospital.

**Fig. 1:**
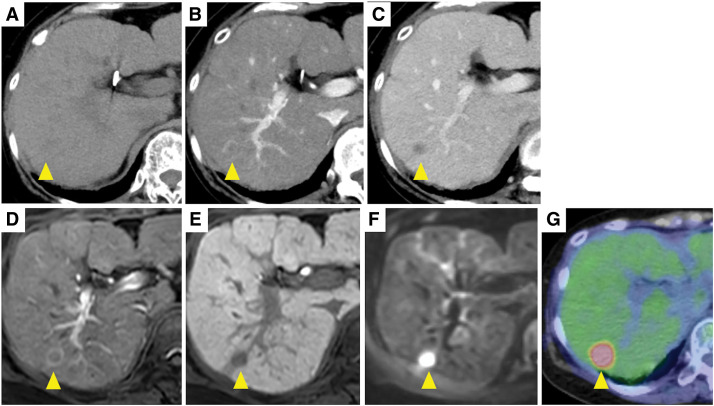
Initial radiological findings. (**A**–**C**) CT demonstrating a 14-mm mass in segment 6 of the liver with peripheral ring-like enhancement. (**D**–**F**) EOB-MRI showing peripheral enhancement, hypointensity in the hepatobiliary phase, and high signal intensity on diffusion-weighted imaging. (**G**) FDG-PET demonstrating increased metabolic activity with SUVmax of 11.2. The arrowheads denote the tumor. EOB-MRI, gadolinium ethoxybenzyl diethylenetriamine pentaacetic acid–enhanced MRI; FDG-PET, fluorodeoxyglucose PET; SUVmax, maximum standardized uptake value

Although malignancy was initially considered, subsequent short-term follow-up demonstrated marked spontaneous regression of the lesion together with decreasing CEA and CA19-9 levels, which strongly suggested an inflammatory lesion, particularly HIPT. In addition, the patient had alcoholic liver cirrhosis with portal hypertension, and careful radiological follow-up was initially selected. This decision was made after discussion among hepatobiliary surgeons, radiologists, and hepatologists at our institutional multidisciplinary conference.

In January of year X–1, follow-up laboratory tests showed a decrease in tumor markers (CEA 3.2 ng/mL and CA19-9 30.0 U/mL). Repeat CT demonstrated spontaneous tumor shrinkage to approximately 5 mm without any therapeutic intervention (**Fig. 2A**–**2C**), and EOB-MRI also confirmed reduction in tumor size (**Fig. 2D**–**2F**). Percutaneous biopsy was considered; however, accurate sampling was considered difficult because the lesion had regressed to approximately 5 mm. In addition, sampling error and bleeding risk related to the background liver disease were taken into account. Given this spontaneous regression, a diagnosis of HIPT was suspected, and close follow-up with EOB-MRI was planned.

**Fig. 2:**
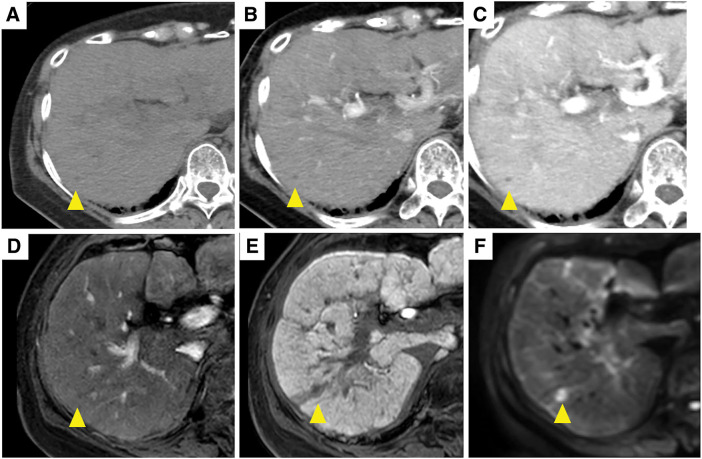
Radiological findings 2 months after initial diagnosis showing spontaneous tumor regression without therapeutic intervention. (**A**–**C**) CT demonstrating marked tumor shrinkage to approximately 5 mm. (**D**–**F**) EOB-MRI confirming reduction in tumor size with decreased conspicuity of the lesion. The arrowheads denote the tumor. EOB-MRI, gadolinium ethoxybenzyl diethylenetriamine pentaacetic acid–enhanced MRI

In April of year X–1, repeat EOB-MRI showed that the lesion remained small at approximately 4 mm, without evidence of progression. Tumor markers also remained stable (CEA 3.2 ng/mL and CA19-9 29.9 U/mL), further supporting the possibility of an inflammatory lesion.

In August and October of year X–1, EOB-MRI revealed gradual tumor enlargement (9 and 12 mm); however, tumor markers remained within the normal range. Surgical resection was reconsidered at that time; however, because the lesion remained relatively small, tumor markers were within normal ranges, and the prior spontaneous regression still favored HIPT, close radiological surveillance was continued.

In February of year X, tumor markers increased again (CEA 5.9 ng/mL and CA19-9 37.8 U/mL), and CT demonstrated tumor enlargement to approximately 20 mm (**Fig. 3A**–**3C**). EOB-MRI confirmed further enlargement (**Fig. 3D**–**3F**), and FDG-PET showed increased metabolic activity (SUVmax 14.3) (**Fig. 3G**). Subsequently, in April of year X, tumor markers rose markedly (CEA 24.9 ng/mL and CA19-9 88.4 U/mL). Given the strong suspicion of malignancy, surgical resection was planned for both diagnostic and therapeutic purposes. Because the patient had alcoholic liver cirrhosis with portal hypertension, preoperative reassessment of liver function and surgical planning were performed before hepatectomy. In May of year X, she underwent laparoscopic partial hepatectomy of segment 6. The postoperative course was uneventful, and the patient was discharged on POD 7.

**Fig. 3:**
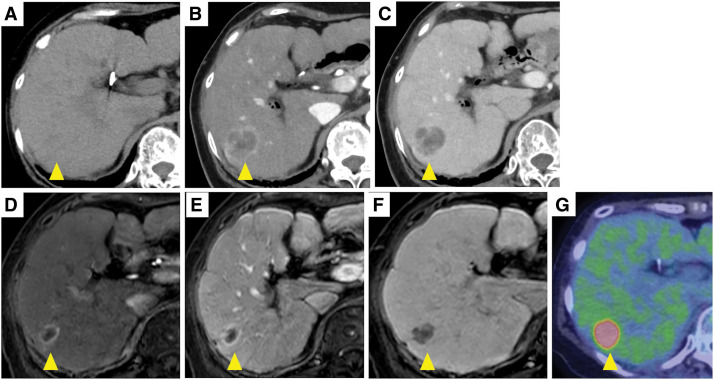
Imaging findings at the time of tumor progression. (**A**–**C**) CT demonstrating tumor enlargement to approximately 20 mm. (**D**–**F**) EOB-MRI confirming progressive enlargement of the lesion. (**G**) FDG-PET showing increased metabolic activity with SUVmax of 14.3. The arrowheads denote the tumor. EOB-MRI, gadolinium ethoxybenzyl diethylenetriamine pentaacetic acid–enhanced MRI; FDG-PET, fluorodeoxyglucose; SUVmax, maximum standardized uptake value

Gross examination of the resected specimen revealed a tumor measuring 46 mm in maximum diameter (**Fig. 4A**). Histologically, invasive growth of the tumor with partial glandular formation and focal mucin production was observed (**Fig. 4B**). Areas of necrosis were occasionally observed (**Fig. 4C**) accompanied by massive neutrophilic infiltration (**Fig. 4D**). Portal vein invasion was identified. Immunohistochemical analysis revealed that tumor cells were diffusely positive for biliary epithelial markers of CK7 and CK19 (**Fig. 4E** and **4F**). Glypican-3 and HepPar-1 were only partially weak positive (**Fig. 4G**, **4H**). Although the tumor had eosinophilic-rich cytoplasm, the overall morphological and immunophenotypic findings excluded the pathological diagnosis of HCC and cHCC-iCCA. Therefore, we made a definitive diagnosis of iCCA with necrotic foci. All surgical margins were negative. Postoperative adjuvant chemotherapy was considered; however, close surveillance was selected because of the patient’s alcoholic liver cirrhosis, portal hypertension, and concerns regarding treatment tolerability.

**Fig. 4:**
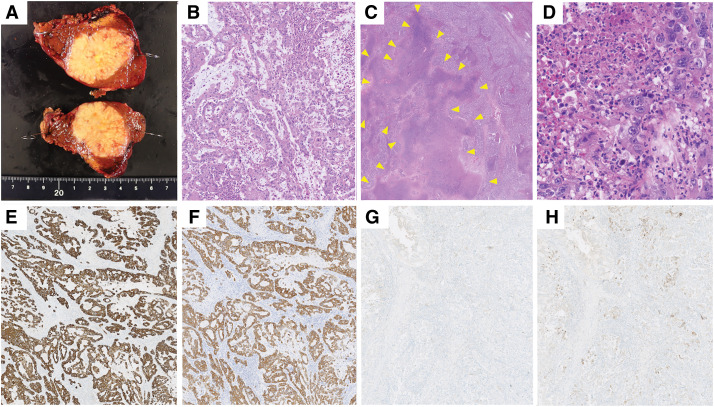
Macroscopic and histopathological findings of the resected specimen. (**A**) Gross specimen showing a whitish-lobulated solid tumor measuring 46 mm in maximum diameter in segment 6. (**B**–**D**) Hematoxylin and eosin staining demonstrating invasive adenocarcinoma with glandular formation and focal mucin production (**B**: original magnification ×100), necrosis (**C**: yellow arrowheads, original magnification ×12.5), and massive neutrophilic infiltration (**D**: original magnification ×400). (**E**–**H**) Immunohistochemical figures (original magnification ×100). The tumor cells are diffusely positive for biliary epithelial markers CK7 (**E**) and CK19 (**F**), they are partially weakly positive for Glypican-3 (**G**) and HepPar-1 (**H**).

At 5 months postoperatively, CT showed no evidence of recurrence. However, 9 months after surgery, tumor markers increased again (CEA 4.6 ng/mL and CA19-9 69.1 U/mL). Subsequent contrast-enhanced CT and FDG-PET/CT revealed a soft tissue mass in the left abdominal wall (SUVmax 10.8) and enlarged para-aortic lymph nodes (SUVmax 5.0), leading to a diagnosis of metastatic recurrence. Systemic chemotherapy with durvalumab plus gemcitabine and cisplatin was initiated. The overall clinical course, including changes in tumor size, serum tumor marker levels, and clinical decision-making during follow-up, is summarized in **Fig. 5**.

**Fig. 5:**
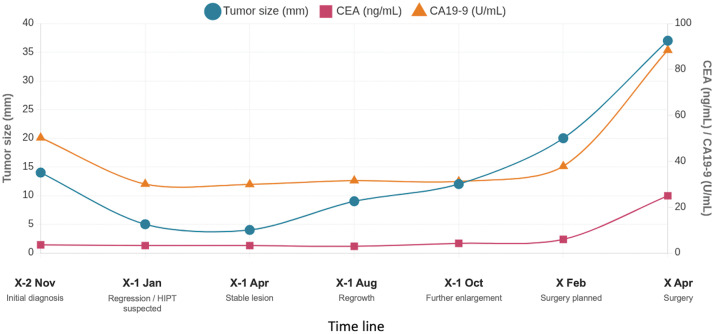
Clinical course of transiently regressed iCCA changes in tumor size, serum tumor marker levels (CEA and CA19-9), and key clinical decision points during follow-up are shown. After initial spontaneous tumor regression with decreased tumor marker levels, HIPT was suspected. However, gradual tumor re-enlargement, re-elevation of tumor markers, and increased FDG uptake during follow-up led to surgical resection, and the final pathological diagnosis was iCCA. CA19-9, carbohydrate antigen 19-9; CEA, carcinoembryonic antigen; FDG, fluorodeoxyglucose; HIPT, hepatic inflammatory pseudotumor; iCCA, intrahepatic cholangiocarcinoma

## DISCUSSION

Spontaneous regression of malignant tumors is defined as partial or complete disappearance of a tumor in the absence of effective therapy. Although spontaneous regression has been reported in several malignancies, it remains exceedingly rare in iCCA.^1,2,9)^ To the best of our knowledge, this is the second reported case. iCCA typically demonstrates aggressive biological behavior, early vascular invasion, and a poor prognosis, making spontaneous tumor regression biologically unexpected.^3)^

Only a limited number of case reports have documented spontaneous or temporary regression of iCCA within the past decade.^5,10)^ In most reported cases, regression was incomplete or transient and was followed by subsequent tumor progression. Proposed mechanisms include tumor ischemia due to vascular alterations, immune-mediated tumor suppression, or inflammatory responses triggered by infection or tissue injury.^5,11)^ These reports suggest that although iCCA predominantly follows a progressive course, spontaneous reduction in tumor burden may occasionally occur under specific biological conditions.

In the present case, histological examination revealed invasive glandular structures with focal mucin production and necrotic areas accompanied by neutrophilic infiltration (**Fig. 4B**–**4D**). Acute inflammatory responses associated with tumor necrosis may activate both innate and adaptive immune pathways through the release of danger-associated molecular patterns, potentially leading to transient suppression of tumor growth or spontaneous regression.^6,7,11)^ However, such immune activation is likely incomplete and temporary; residual viable tumor cells may persist and subsequently proliferate once the inflammatory response subsides. This dynamic process may explain the biphasic clinical course observed in this case, characterized by initial regression followed by tumor regrowth. Furthermore, the desmoplastic tumor microenvironment characteristic of iCCA may limit sustained immune-mediated tumor eradication and facilitate tumor re-expansion.^1,3)^

A major diagnostic challenge in this case was differentiation from HIPT, a benign inflammatory mass composed of fibroinflammatory tissue and inflammatory cell infiltration.^12,13)^ HIPT frequently mimics malignant liver tumors on imaging, including iCCA, because both entities can exhibit peripheral ring enhancement on contrast-enhanced CT or MRI and increased uptake on FDG-PET.^12–14)^ Importantly, spontaneous regression is well documented in HIPT and is considered one of its characteristic clinical features.^13)^ Despite these similarities, several findings in the present case were atypical for HIPT. First, FDG uptake was relatively high (SUVmax >10), which more commonly suggests malignancy, although it is not specific. Second, CEA and CA19-9 re-elevated during follow-up, which is less typical for benign inflammatory lesions. Third, progressive enlargement after initial shrinkage suggested persistent malignant potential rather than resolving inflammation. In the present case, the marked spontaneous regression and normalization of tumor markers initially favored an inflammatory diagnosis and contributed to the decision to defer immediate surgery.

The differential diagnosis between iCCA and HIPT remains challenging. Imaging findings overlap substantially, and no single modality reliably distinguishes them in all cases. IgG4-related HIPT may further complicate diagnosis because it can closely resemble iCCA both radiologically and clinically.^6)^ Although percutaneous biopsy may aid diagnosis, sampling error and procedure-related risks should be considered.^15)^ In addition, because the patient had underlying alcoholic liver cirrhosis with portal hypertension, the extent of liver resection was intentionally minimized to preserve remnant liver function. In the present case, progressive tumor growth and rising tumor markers justified surgical resection, and pathological examination confirmed invasive iCCA with portal vein invasion. Negative surgical margins were achieved.

This case underscores an important clinical message: spontaneous tumor regression does not exclude malignancy. When radiologic suspicion persists or tumor markers trend upward, continued surveillance and timely surgical intervention should be considered even in the setting of temporary tumor shrinkage.

From a biological perspective, transient tumor regression may reflect short-term immune control rather than true tumor eradication. The immunosuppressive tumor microenvironment characteristic of iCCA may limit durable anti-tumor responses and permit subsequent tumor regrowth.^1,3)^ Further investigation into the tumor-immune dynamics in iCCA may provide insights into mechanisms of tumor dormancy and reactivation, as well as potential therapeutic strategies.

## CONCLUSIONS

We present a rare case of iCCA that transiently regressed without treatment, mimicked HIPT, and ultimately progressed, necessitating surgical intervention. This case highlights that spontaneous tumor regression does not exclude malignancy, and multidisciplinary evaluation and careful surveillance are warranted when iCCA is in the differential diagnosis.

## DECLARATIONS

### Availability of data and materials

All data generated or analyzed during this study are included in this published article.
